# Protein–protein interactions and the spatiotemporal dynamics of bacterial outer membrane proteins

**DOI:** 10.1016/j.sbi.2015.10.007

**Published:** 2015-12

**Authors:** Colin Kleanthous, Patrice Rassam, Christoph G Baumann

**Affiliations:** 1 Department of Biochemistry, University of Oxford, South Parks Road, Oxford OX1 3QU, UK; 2 Department of Biology, University of York, York YO10 5DD, UK

## Abstract

It has until recently been unclear whether outer membrane proteins (OMPs) of Gram-negative bacteria are organized or distributed randomly. Studies now suggest promiscuous protein–protein interactions (PPIs) between β-barrel OMPs in *Escherichia coli* govern their local and global dynamics, engender spatiotemporal patterning of the outer membrane into micro-domains and are the basis of β-barrel protein turnover. We contextualize these latest advances, speculate on areas of bacterial cell biology that might be influenced by the organization of OMPs into supramolecular assemblies, and highlight the new questions and controversies this revised view of the bacterial outer membrane raises.

## Highlights

• We discuss spatiotemporal patterning in the bacterial outer membrane.
• Promiscuous interactions between outer membrane proteins govern their behaviour.
• Turnover and biogenesis of outer membrane proteins linked to formation of clusters.
• Implications of spatiotemporal patterning for bacterial physiology discussed.

**Current Opinion in Structural Biology** 2015, **35**:109–115

This review comes from a themed issue on **Protein**–**protein interactions**

Edited by **Alexandre M J J Bonvin** and **Özlem Keskin**

For a complete overview see the Issue and the Editorial

Available online 26th November 2015

**http://dx.doi.org/10.1016/j.sbi.2015.10.007**

0959-440/© 2015 The Authors. Published by Elsevier Ltd. This is an open access article under the CC BY license (http://creativecommons.org/licenses/by/4.0/).

## Introduction

Gram-negative bacteria need a stable outer membrane (OM) to colonise diverse environments such as soil and water, and animals and humans where they can be both commensals and pathogens [1^•^]. One reason Gram-negative bacteria are so robust, adaptable and naturally resistant to antibiotics such as vancomycin is their unique OM, an asymmetric bilayer composed of an inner leaflet of phospholipids and an outer leaflet of lipopolysaccharide (LPS). LPS, which is essential in most Gram-negative bacteria, is further stabilized by divalent cations (Mg^2+^, Ca^2+^) that form non-covalent cross-bridges between adjacent molecules.

A consequence of bacteria having an OM is that many secreted proteins (OMPs and lipoproteins) are needed to support its functions such as biogenesis of membrane components, maintenance of OM integrity, nutrient uptake, export of waste products, cell adhesion, evasion of host defenses and virulence. OMPs range in size (from 8 to 26 β-strands), oligomeric structure (monomers to trimers) and copy number (from a few hundred to hundreds of thousands of copies per cell). Following secretion through the Sec translocon, unfolded OMP polypeptides are delivered to the β-barrel assembly machine (BAM) by periplasmic chaperones for insertion into the OM. The BAM complex is composed of an OMP (BamA), which catalyses OMP insertion, and four accessory lipoproteins (BamBCDE) [2•, 3•]. BamA is essential and highly conserved in Gram-negative bacteria. Related molecular machines are found in mitochondria and chloroplasts, eukaryotic organelles evolved from intracellular bacteria, which also have β-barrel proteins in their outer membranes [4, 5].

Over the last 10 years there has been significant progress in our understanding of the molecular mechanism of OMP biogenesis, including discovery of the BAM complex [6^••^], structure determination of BamA [7^••^] and *in vitro* reconstitutions of BamA-catalysed OMP folding [8••, 9••]. By contrast, what happens to OMPs after folding is less well understood. Recently, however, with the advent of novel OMP labelling strategies coupled with advances in imaging techniques has come new insight into what happens to folded OMPs once inserted in the OM of bacteria, principally the model organism *E. coli*. We highlight these developments, place them in the context of previous biophysical measurements of OMP mobility and localization data, and discuss the physiological implications of supramolecular OMP assembly.

## OMPs have restricted mobility in the OM of Gram-negative bacteria

The mobility of fluorescently labelled OMPs in bacterial cells has generally been studied through two approaches, fluorescence recovery after photobleaching (FRAP) in confocal microscopy and single-particle tracking (SPT) in total internal reflection fluorescence (TIRF) microscopy. See Table 1 for a summary of published SPT studies on OMP mobility data. FRAP-based studies have yielded conflicting results. FRAP analysis of *E. coli* OMPs randomly labelled with a maleimide Alexa dye suggested recovery of fluorescence in <1 min [10], although this approach cannot discount the labelling of periplasmic proteins. Conversely, FRAP experiments using specifically labelled OMPs indicate they are immobile on long timescales. Verhoeven *et al*. [11], using a mCherry-OmpA fusion, showed absence of FRAP even after 15 min, and that removal of the peptidoglycan-binding domain of OmpA did not influence this behaviour. Rassam *et al*. [12^••^] conducted FRAP experiments on *E. coli* cells using fluorescently-labelled colicins, ColE9 and ColIa, which bind with high affinity to the vitamin B_12_ transporter, BtuB, and the iron siderophore transporter, Cir, respectively. In both cases no FRAP was observed after 3 min in confocal microscopy experiments. This study also found that *E. coli* cells devoid of major cell envelope structures/processes (porins, proton motive force (pmf), TolA, Pal, TonB, Braun's lipoprotein Lpp and with truncated LPS) also did not result in FRAP in confocal experiments. In summary, current evidence suggests the long-range immobility of OMPs is not due to interactions with the underlying cell wall or trans-envelope systems coupled to the pmf.

While the picture emerging from FRAP experiments is that OMPs cannot diffuse across the entirety of the OM, single molecule experiments indicate OMPs exhibit local diffusion. Tracking of individual LamB by a variety of approaches (colloidal gold labelling in differential interference contrast microscopy, attachment of streptavidin-coated polystyrene beads in an optical trap, streptavidin conjugated fluorophores/quantum dots or eYFP-lambda-phage in SPT experiments) generally show that diffusion of this OMP is Brownian on short-timescales but confined on longer time-scales, and its distribution in the OM is heterogeneous [13•, 14, 15]. Spector *et al*. [16] used SPT to show both BtuB, a low abundance monomeric OMP (∼200–300 copies/cell), and OmpF, an abundant trimeric OMP (∼1 × 10^5^ monomers/cell) undergo restricted diffusion. Similarly, Rassam *et al*. [12^••^] found BtuB and Cir exhibited restricted diffusion in SPT-TIRFM experiments (Table 1).

Collectively, membrane diffusion data of OMPs in live *E. coli* cells indicate they generally display somewhat slower diffusion coefficients than those of inner membrane proteins (∼0.1–0.01 μm^2^/s). See reference [17] for a more detailed review of bacterial membrane diffusion studies. Where the diffusion of OMPs differs significantly from most inner membrane proteins is in their confinement to regions of the membrane, estimated from the various published studies to be 0.03–0.60 μm confinement diameter. This confinement readily explains why fluorescently labelled OMPs show no recovery of fluorescence in FRAP experiments. Analysis of restricted diffusion in membranes sometimes reveals anomalous subdiffusion, where the mean-squared displacement (MSD) in time is non-linear and characterised by an exponent *α* < 1 (Table 1). Membrane subdiffusion can occur due to transient immobilisation, for example, induced by PPIs, protein–lipid interactions or non-interacting physical barriers [18, 19].

## Promiscuous protein–protein interactions restrict the lateral diffusion of OMPs

**Figure 1:**
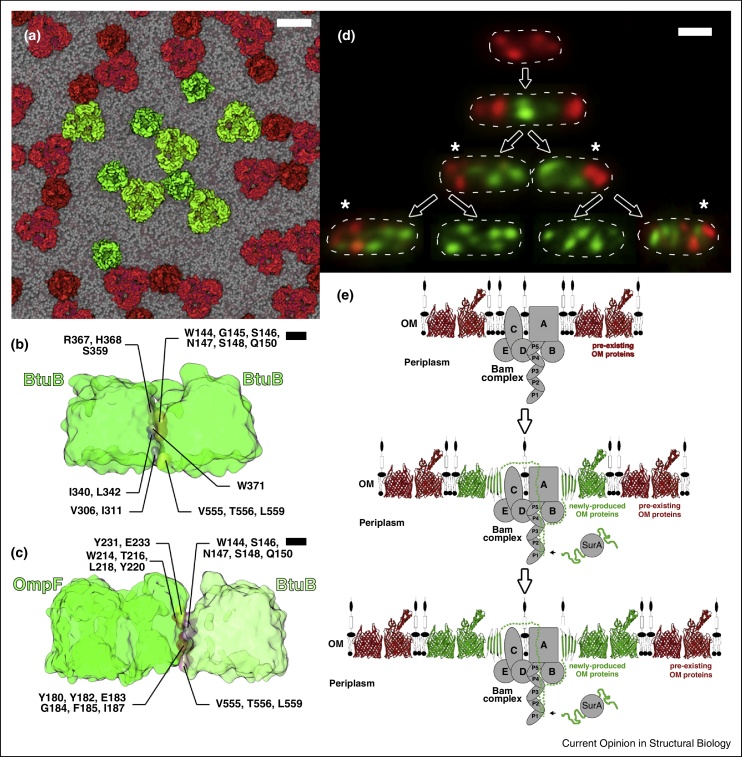
Promiscuous protein-protein interactions contribute to spatiotemporal patterning of the *E. coli* outer membrane. **(a)** Top view of promiscuous protein–protein interactions between monomeric BtuB and trimeric OmpF from a 10 μs coarse grained MD simulation in a PE:PG (3:1) bilayer (see [12^••^] for further details). Green and red labels are used merely to illustrate how clusters of OMPs (red) might exclude other clusters (green) through networks of interactions. We suggest such networks may be the basis for the formation of supramolecular assemblies of OMPs, with intervening LPS (not present in this simulation) separating OMP assemblies. Scale bar corresponds to 5 nm. **(b)** and **(c)** Lateral views of promiscuous BtuB–BtuB and BtuB–OmpF interactions from MD simulations highlighting residues (mainly hydrophobic) at the interfaces of these complexes. Scale bar corresponds to 1 nm. **(d)** Composite TIRFM images of OMP islands (see [12^••^] for further details) in which the vitamin B_12_ receptor BtuB was stained with fluorescently-labelled colicin E9. New OMP islands (labelled with AlexaFluor 488, green label) emerge around midcell pushing old OMP islands (labelled with tetramethylrhodamine, red label) towards the poles. The resulting binary partitioning of OMPs generates repository cells (asterisk) containing most of the original old OMPs. Daughter cells with completely new OMPs emerge after two generations. Scale bar corresponds to 1 μm. **(e)** Schematic showing the sequential insertion of newly synthesized OMPs (green) in the outer membrane by the BAM complex (grey), via unfolded OMPs bound to a periplasmic chaperone (SurA), pushing pre-existing old OMPs (red) outwards in conjunction with cell elongation.

An increasing number of studies point to promiscuous PPIs between OMPs and the formation of large OMP clusters as the basis for their characteristic diffusion behaviour in the OM of *E. coli*. AFM studies have highlighted tight intermolecular packing of OMPs in the OM of Gram-negative bacteria [20•, 21] as well as in the OM of mitochondria [22], which also have an abundance of OMPs. High speed AFM studies of OmpF reconstituted in supported bilayers at high surface densities uncovered a complex pattern of diffusive properties ranging from freely mobile trimers to immobile clustered aggregates [23^••^]. Moreover, an ‘interaction map’ of individual OmpF trimers from accompanying coarse-grained molecular dynamics (MD) simulations showed qualitative agreement with the interactions revealed by the AFM data [23^••^]. Other coarse-grained MD simulations also point to OMPs having a propensity for self-association in membranes [24]. In the case of BtuB, MD simulations indicate the same bulky hydrophobic residues displayed from the intramembrane regions of the β-barrel mediate both BtuB–BtuB and heterologous BtuB–OmpF associations [12^••^] (Figure 1a–c). The promiscuous PPIs of OMPs observed in membranes are generally not manifest in purified preparations of the same proteins (e.g. BtuB) presumably because detergents commonly used to solubilize OMPs for biochemical and structural analysis mask the interacting regions involved.

Rassam *et al*. [12^••^] recently provided compelling evidence that promiscuous PPIs between OMPs explains their characteristic diffusion in the OM of *E. coli*. They found that BtuB labelled with fluorescently labelled colicin E9 and reconstituted in a supported phospholipid bilayer *in vitro* exhibited diffusion characteristics (lateral diffusion coefficient and confinement diameter) similar to BtuB in the OM of *E. coli* (Table 1). This effect could be elicited merely by raising the BtuB concentration or by adding another OMP at high concentration (OmpF) to the bilayer, but not by the addition of a non-barrel protein.

## Spatiotemporal patterning of OMPs in the Gram-negative outer membrane

Several studies suggest the distribution of OMPs in the OM of Gram-negative bacteria is not homogeneous, with various patterns observed depending on the method of observation and the spatial resolution of the experiments. de Pedro *et al*. [25] recorded growth-dependent patterning of the *E. coli* OM by fluorescence microscopy following random labelling with a covalent fluorophore, the partitioning to the poles interpreted as evidence of peptidoglycan-directed movement of OMPs. Using fluorescent phage binding to LamB in live-cell epifluorescence microscopy, Gibbs *et al*. [14] observed fluorescence that was either bipolar or patchy with regular and irregular spiral patterns. Rothenburg *et al*. [15] observed patchy fluorescence of LamB molecules as well as circumferential rings and single/double helices. Ursell *et al*. [26^••^] used specific labelling of overexpressed LamB (via a ybbr tag inserted into a surface exposed loop) in fluorescence microscopy experiments to demonstrate random, burst-like appearance of LamB on the surface of *E. coli* that moved to the poles during growth. Earlier EM studies by Smit and Nikaido [27] using ferritin-labelled antibody found *Salmonella* OmpF also appeared in patches in the OM.

More recently, TIRFM data of colicin-labelled BtuB and Cir revealed these OMPs co-localize within large clusters called OMP islands [12^••^] (Figure 1d). BamA (labelled with antibody) was also found within OMP islands suggesting that once inserted in the OM folded OMPs do not diffuse far from the biogenesis machine that deposited them there (Figure 1e). The average size of an OMP island was ∼0.5 μm, consistent with these micro-domains containing hundreds (possibly thousands) of OMPs (estimated mass >50 MDa). Rassam *et al*. [12^••^] speculated that since the confinement diameter observed for OMP diffusion *in vitro* is similar to the average size (and confinement diameter) of an OMP island *in vivo*, the restricted diffusion and patterning of OMPs in the Gram-negative OM is governed by promiscuous interactions between OMPs.

The studies of Ursell *et al*. [26^••^] and Rassam *et al*. [12^••^] demonstrate that OMP islands move to the poles as cells grow and this movement is driven by new OMP biosynthesis. Although OMP biogenesis occurs predominantly at mid-cell the appearance of new OMP islands is stochastic and can occur anywhere on the cell surface except at the poles (Figure 1d).

A ‘chicken-and-egg’ problem for bacteria arises with the organization of OMPs into large clustered islands: If BAM is contained within OMP islands and BamA is itself a β-barrel, how do new OMP islands emerge? A potential solution to this problem comes from recent reports showing BAM complex accessory proteins, all of which are lipoproteins, promote insertion of BamA in the membrane [28•, 29]. This would imply β-barrel assembly in the OM begins via the lipoprotein (Lol) pathway [30] although this has yet to be formally demonstrated.

## Binary OMP partitioning — a new mechanism for protein turnover

How OMPs change from one generation to the next especially in response to changes in environmental conditions is not understood. This is an important problem since the expression of many of the >100 OMPs encoded by the *E. coli* genome are tightly regulated. Moreover, some OMPs are present at high copy number making ‘dilution-through-division’ an inefficient means of turnover. A further compounding factor is the very high stability of OMPs, which typically exhibit folding free energies in excess of −20 kcal/mol [31]. Yet the OM lacks an energy source through which OMPs could be extracted and degraded, as happens with inner membrane proteins by the ATP-dependent protease FtsH [32].

Spatiotemporal organization explains how OMPs can be turned over rapidly in bacteria without the need to degrade them. The strict spatial segregation of old (at the poles) and new (primarily at mid-cell) OMP islands means septation results in a binary distribution of OMPs [12^••^]. The division of every *E. coli* cell then generates repository cells in which old OMPs from the preceding mother cell are housed (Figure 1d). As a consequence of binary OMP partitioning, cells with completely new OMPs appear after only two generations.

## Implications of the spatiotemporal organization of OMPs

The organization of OMPs into islands and their segregation through binary partitioning has the potential to influence several aspects of bacterial cell envelope biology:

(1) *OMP memory*. The persistence of old OMPs at the poles of rod-shaped bacterial cells could endow populations with phenotypic heterogeneity reflecting memory of past growth conditions. Such an epigenetic mechanism could buffer against fluctuations in the concentrations of scarce nutrients, influence susceptibility of bacterial populations to antibiotics and phage infection and modulate the sensitization of bacterial cells towards the immune system of a host.
(2) *Polar localization*. Several mechanisms have been documented that result in the asymmetric distribution of cytoplasmic proteins to the poles of cells, which is important for cell division, chemotaxis and virulence [33, 34]. The flow of OMP islands to the poles of dividing *E. coli* cells under the force of OMP biogenesis represents a new mechanism for polar localization of proteins. The longitudinal movement of OMP islands implies *all* OMPs will by default end up at cell poles.
(3) *Ageing*. Symmetrically dividing bacteria such as *E. coli* are thought to undergo ageing; cells inheriting very old poles are less fit than their new pole counterparts [35, 36]. Current theories suggest asymmetric segregation of damaged cytoplasmic proteins could be the cause of ageing in bacteria [37]. We suggest retention of old OMPs at the poles of cells, exposed for long periods of time to damage by oxidation and proteolysis, could also be a contributory factor to cellular ageing in bacteria.
(4) *Coordination of outer membrane processes*. Spatiotemporal organization of OMPs could coordinate processes in the OM required for its integrity and maintenance. The OMP LptD, in conjunction with the lipoprotein LptE, inserts LPS into the outer leaflet of the OM and is essential in most Gram-negative bacteria [38]. LptD is a β-barrel [39, 40] and hence a BamA substrate so it is conceivable that LPS and OMP biogenesis are coordinated by virtue of their co-localization within OMP islands. Indeed, there is close genetic linkage between the LPS and OMP biogenesis pathways [6^••^], and LPS is known to facilitate the assembly of trimeric OMPs [41]. OM lipoprotein and OMP biogenesis could also be coordinated through co-localization in OMP islands. Rassam *et al*. [12^••^] detected BamC, one of the accessory lipoproteins of BamA, in OMP islands. Furthermore, the periplasmic lipoprotein RcsF, which senses cell envelope stress, is partly exposed on the surface of *E. coli* via the central lumen of OMPs (OmpF, OmpC and OmpA) [42•, 43•]. Given its close associations with different OMPs, RcsF (and possibly other lipoproteins) might reside within OMP islands.

## Outstanding questions and controversies

Spatiotemporal organization of OMPs in *E. coli* raises many new questions about the OM: Is similar organization observed in other rod-shaped bacteria? How does OMP turnover compare between rod-shaped and spherical Gram-negative bacteria? Where/how is LPS distributed in the OM and how much is present within OMP islands? Does LPS account for the lack of intermixing of old/new OMP islands? What causes the apparent inhibition of further OMP biogenesis within old OMP islands? Are abundant and rare OMPs co-localized within the same OMP islands? Do OMPs of different size and oligomeric structure pack together within OMP islands? If so, how does this affect the morphology of OMP islands? Is the confinement of OMPs when reconstituted in supported bilayers indicative of OMP island formation? If so, what governs the size limitation of the islands?

Many β-barrel autotransporter proteins (e.g. IcsA, BimA) are localized directly at the poles of Gram-negative bacteria [44, 45], raising the question of whether their mechanism of insertion in the OM is distinct to that of other OMPs that move to the poles as part of OMP islands. Autotransporters display or release passenger domains at the cell surface and serve important functions in bacterial pathogenesis, including assembling actin tails for intracellular transport (reviewed in [46]). The direct targeting of autotransporters to the cell pole begins in the cytoplasm [45, 47] and requires BamA for insertion of their β-barrel domains in the OM [48^•^]. Yet the work of Rassam *et al*. [12^••^] suggests the BAM complex is inactive (at least for BtuB and Cir biogenesis) when localized at the poles. Might this provide an explanation for the involvement of the translocation and assembly module (TAM) complex, which spans the cell envelope and is involved in autotransporter biogenesis [49] but the role of which remains enigmatic? Finally, work on IcsA in *Shigella flexneri* suggests that after polar localization the protein moves towards midcell [50], which is in the opposite direction to the bulk flow of OMPs to the poles observed in *E. coli*. Further work will be needed to reconcile these issues.

## Conflict of interest

Nothing declared.

## References and recommended reading

Papers of particular interest, published within the period of review, have been highlighted as:

• of special interest
•• of outstanding interest

## Figures and Tables

**Table 1:** Comparison of 2D diffusive behaviour for OMPs determined using different SPT methods

Protein^a^ (*E. coli* strain or PSM)	SPT method^b^ (imaging rate)	SPT probe^c^	Brownian diffusion (MSD = 4*Dt*)		Anomalous subdiffusion (MSD = 4*Dt*^*α*^)	Reference
			*D* (μm^2^/s)	Confinement diameter (μm)	Exponent^f^ (α)	
LamB^biotin^ (S2188:pLO16)	BFM (25 Hz)	0.53 μm SA-PS bead held in optical tweezers	0.15	0.03	NR	[13^•^]
LamB^Au-BE^ (*lamB*^–^)	DICM (1 Hz)	20 nm colloidal gold	NR^d^	NR^e^, < 0.2	NR	[14]
LamB^Au-BE^ (*lamB*^+^)	DICM (1 Hz)	20 nm colloidal gold	NR^d^	NR^e^, < 0.2	NR	[14]
LamB (LE392)	FM (30 Hz)	eYFP-λ phage particle	0.059	NR, < 0.4	0.3	[15]
LamB^biotin^ (*lamB*^–^)	FM (30 Hz)	SA–Qdot	0.058	NR, < 0.4	NR	[15]
BtuB (K17)	FM (40 Hz)	^AF555^Antibody	0.05	NR	0.56	[16]
BtuB (K17)	FM (40 Hz)	^OG488^Colicin E3	0.1	NR	0.75	[16]
BtuB^TonB^^box^^mutant^ (*btuB*^–^)	FM (40 Hz)	^AF555^Antibody	0.27	NR	0.56	[16]
BtuB (JM83)	FM (30 Hz)	^AF488^Colicin E9^S-S^	0.013	0.6	0.53	[12^••^]
BtuB (PSM ^BtuB^^1000x^)	FM (30 Hz)	™^R^Colicin E9^S-S^	0.013	0.6	0.34	[12^••^]
BtuB (PSM ^BtuB^^1x:^^OmpF^^1000x^)	FM (30 Hz)	™^R^Colicin E9^S-S^	0.012	0.6	0.51	[12^••^]
BtuB (JM83)	FM (56 Hz)	^AF488^Colicin E9^S-S^	0.025 (*N* = 54)	0.5	0.62	unpublished
BtuB (JM83)	FM (30 Hz)	^AF488^Δ^1-52^Colicin E9^S-S^	0.0081	0.5	0.64	[12^••^]
BtuB (BZB1107)	FM (30 Hz)	^AF488^Colicin E9^S-S^	0.018	0.5	0.11	[12^••^]
Cir (JM83)	FM (30 Hz)	^AF488^Colicin Ia^S-S^	0.019	0.6	0.20	[12^••^]
Cir (BZB1107)	FM (30 Hz)	^AF488^Colicin Ia^S-S^	0.011	0.5	0.86	[12^••^]
OmpF (K17)	FM (40 Hz)	^AF555^Antibody	0.006	0.1	0.14	[16]

a Au-BE denotes gold-binding epitope. PSM denotes *in vitro* polymer-supported membrane containing reconstituted BtuB and/or OmpF (see [12^••^] for experimental details).

b BFM denotes bright-field microscopy. DICM denotes differential interference contrast microscopy. FM denotes fluorescence microscopy.

c AF488, AF555, OG488 and TMR denote the following fluorescent dyes (respectively): Alexa Fluor 488, Alexa Fluor 555, Oregon Green 488 and tetramethyl rhodamine. S–S denotes disulphide top-lock in R-domain to prevent colicin translocation across the bacterial outer membrane.

d Motion of colloidal gold was tracked for 5 min at 1 Hz. Immobile (20–50 nm displacement) and somewhat mobile (100–300 nm displacement) particles were observed in both the presence and absence of wild-type LamB.

e Confinement diameter was estimated from the asymptotic MSD value at 10 s. This parameter was not reported in Ref. [14].

f This exponent term (*α*) describes the non-linear scaling of the MSD in time, with *α* < 1 indicating anomalous subdiffusion and *α* = 1 indicating normal Brownian diffusion.
